# Evaluation of pre-processing methods for tear fluid proteomics using proximity extension assays

**DOI:** 10.1038/s41598-023-31227-1

**Published:** 2023-03-17

**Authors:** Daphne P. C. Vergouwen, Amber J. Schotting, Tanja Endermann, Harmen J. G. van de Werken, Dwin G. B. Grashof, Sinthuja Arumugam, Rudy M. M. A. Nuijts, Josianne C. ten Berge, Aniki Rothova, Marco W. J. Schreurs, Marlies Gijs

**Affiliations:** 1 Department of Ophthalmology, Erasmus MC, University Medical Center Rotterdam, Rotterdam, The Netherlands; 2 Department of Immunology, Erasmus MC, University Medical Center Rotterdam, Rotterdam, The Netherlands; 3 Olink Proteomics, Uppsala, Sweden; 4 University Eye Clinic Maastricht, School for Mental Health and Neuroscience (MHeNs), Maastricht University, Maastricht, The Netherlands

**Keywords:** Biomarkers, Biomarkers, Translational research, Proteomic analysis

## Abstract

Tear fluid forms a potential source for biomarker identification, and can be minimal invasively collected via Schirmer strips. The lack of knowledge on the processing of Schirmer strips however complicates the analysis and between-study comparisons. We studied two different pre-processing methods, specifically the use of punches of the strip versus elution of the strip in a buffer. Tear fluid filled Schirmer strips were collected from 5 healthy participants, and divided into two halves over the length of the strip. In either part, punches or eluates were obtained from 4 different locations, from the first part touching the eye (head) to the end, to assess the protein distribution along the strips. The levels of 92 inflammatory proteins were measured in the punches/eluates using proximity extension assays. The punch method yielded higher protein detectability compared to the elution method (76% vs 66%; p ≤ 0.001). Protein expression level was found to be slightly higher in the head of the strip, however, 3 out of 5 punches from the head failed quality control. Protein expression levels over the remaining parts of the strips were similar. Our study showed beneficial use of punches of any part of the strip except the head in future biomarker research.

## Introduction

Tear fluid is a crucial component of the human eye, even though it represents a layer of only a few microns thick. It has an important refractive function, provides oxygen and electrolytes to the cornea, offers smooth eye lid movement and protects the ocular surface from environmental factors. The latter is accomplished through regulated secretion of protective factors, including hydrating glycoproteins, antimicrobials, wound healing factors, and anti-inflammatory proteins^1,2^.

Dysfunction of tear fluid production or stability can lead to decreased visual acuity and complications^3^. Various (inflammatory) eye conditions or systemic disorders may lead to altered tear fluid composition. Given the close contact to the eye, and its highly abundant and concentrated proteome, changes in tear fluid composition may reflect pathogenic mechanisms^4–6^. At present there is an increasing interest in using tear fluid samples to investigate ocular and systemic diseases^2,4,7–13^. Multiplex protein technologies are preferred for its analyses, due to the low sample volume required and high sensitivity. For example by addressable laser bead immunoassay (Luminex), electrochemiluminescence (Meso Scale Discovery), and proximity extension assays (PEA). PEA enables analysis of hundreds of analytes simultaneously with high specificity and relative protein quantification.

Tear fluid can be collected using various methods, of which Schirmer strips and capillaries are the most common^4^. Tear fluid collection by Schirmer strips is easy to perform, well-accepted by patients, and yields the highest protein content^5,14,15^. As a consequence, the majority of studies investigating tear proteins use these strips^16–18^. However, the handling of Schirmer strips for subsequent protein analysis varies greatly, and knowledge on the protein behaviour on Schirmer strips is lacking^2,19,20^. Standardization is essential to compare studies and allow further research into potential biomarkers for personalized medicine.

In this study we aim to optimize the use of Schirmer strips for tear fluid protein analysis using PEA technology. We will compare two different pre-processing methods of tear fluid Schirmer strips (punch versus elution). Furthermore, we examine the protein composition and migration along the strip area.

## Results

### Protein detectability using two pre-processing methods (punch versus elution)

**Figure 1:**
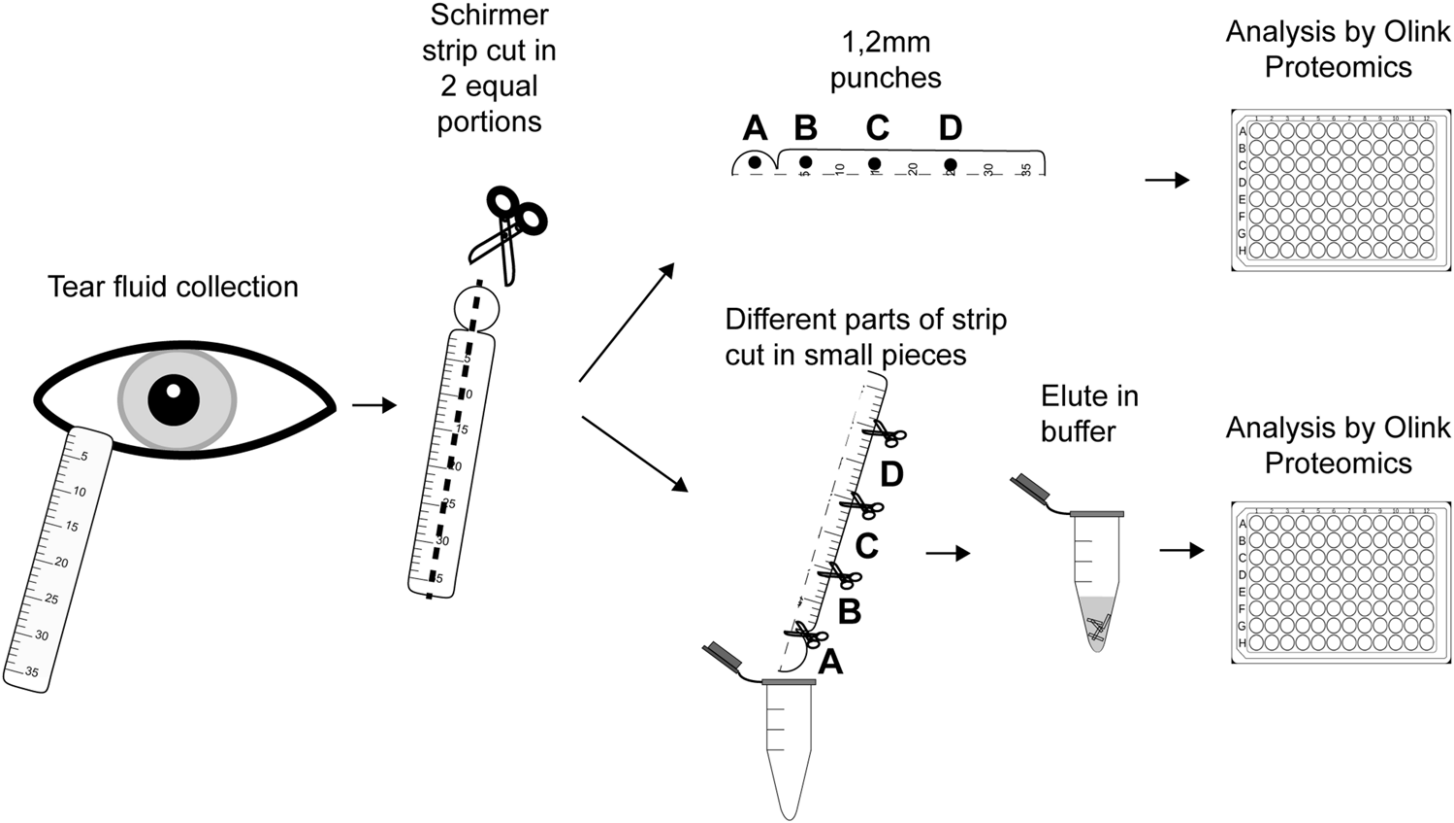
Experimental set-up for comparison of tear fluid pre-processing methods, and protein distribution on a Schirmer strip using PEA technology by Olink Proteomics. Tear fluid from 5 healthy volunteers was collected with Schirmer strips. Strips were cut vertically into two equal portions. Out of one part of the strip, four 1.2 mm punches were taken from various locations of the strip (A: head; B: 0–10 mm; C: 10–20 mm; D: 20–30 mm). The other part of the strip was cut into horizontal locations (A: head; B: 0–10 mm; C: 10–20 mm; D: 20–30 mm) and subsequently the locations were eluted in 60 µL buffer. The punches as well as 1 out of 40 µl of the eluates were supplied to Olink Proteomics in a 96 wells plate.

**Figure 2:**
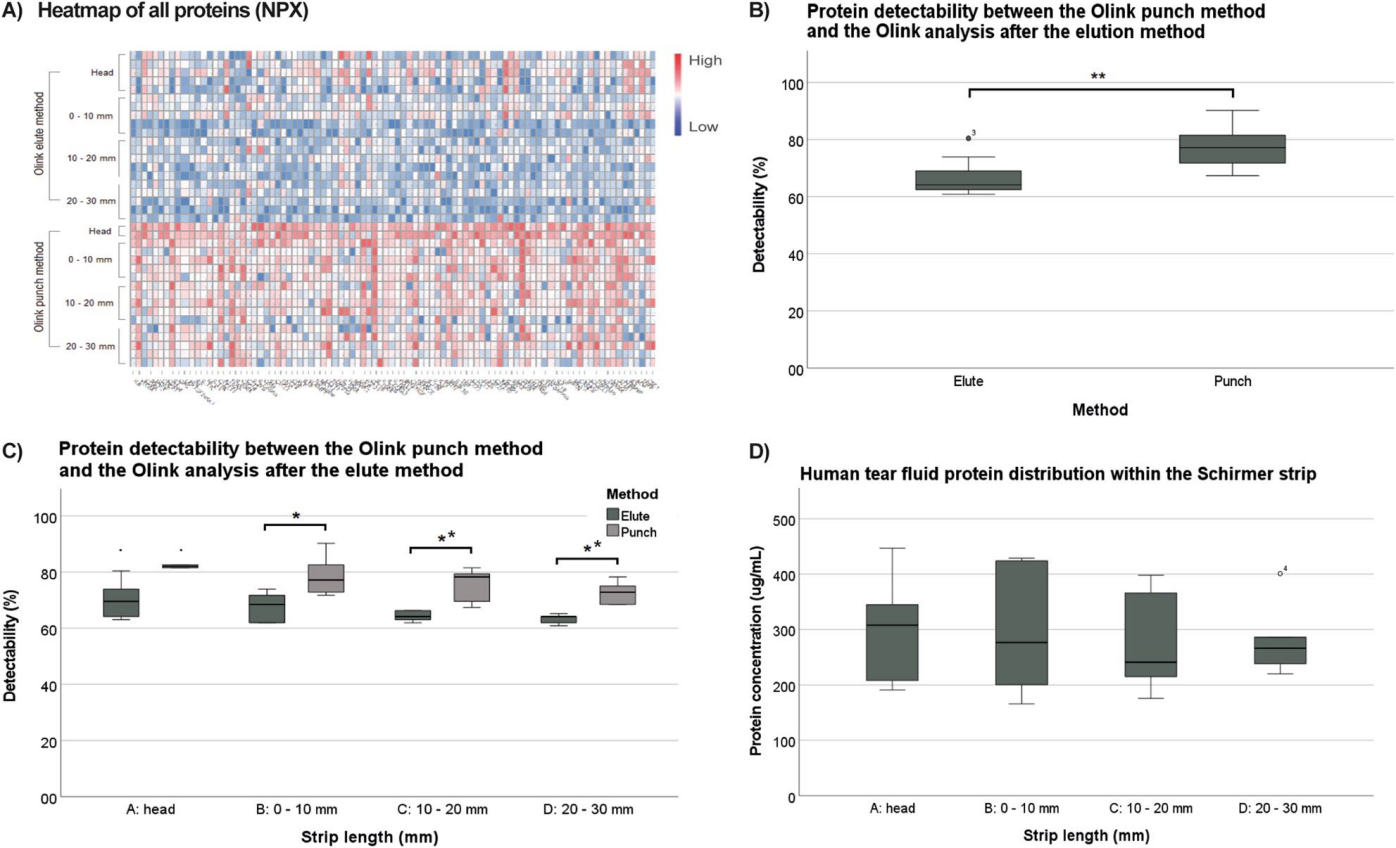
Protein detectability using the punch method versus elution of tear fluid Schirmer strips. (**A**) Heatmap of protein expression level (normalized protein expression (NPX)) of 92 proteins tested with the Olink Target 96 panel. Individual proteins are depicted on the X-axis, while samples of the different pre-processing methods and locations are shown on the Y-axis. (**B**) The boxplots depict the protein detectability (% of proteins of a total number of 92 proteins above the lower limit of detection) between the punch and elution method. Overall, protein detectability is significantly higher in the punch method compared to the elution method (p = 0.002). (**C**) Protein detectability of different locations of the strip using the punch method (A: 82 ± 0.8%; B: 79 ± 7.6%; C: 75 ± 6.3%; D: 73 ± 4.2%) and the elution method (A: 70 ± 7.2%; B: 68 ± 5.5%; C: 64% ± 1.9%; D: 63 ± 1.8%) showed significant differences (A: p = –; B: p = 0.028; C: p = 0.006; D: p = 0.002). Protein detectability was not significantly different between the locations of the strip for the elute method (p = 0.122) as well as for the punch method (p = 0.231). Three out of twenty Schirmer strip pieces did not pass the quality control and were excluded in 2A, 2B, and 2C. These three Schirmer strip pieces were all from location A of the punch method. (**D**) The boxplot depicts the total protein concentration found in the different Schirmer strip locations. Total protein concentration, measured by the bicinchoninic acid assay (BCA), was similar between all locations of the Schirmer’s strip. Surface area difference of location A vs the other locations of the strip was corrected for with the correction factor = 4/π.

The experimental set-up is shown in Fig. 1. In the analysis 92 inflammatory proteins were tested, that might be of importance in future biomarker research. The tear fluid expression of included proteins is yet partly unknown. From the total of 40 samples, three did not pass the Olink quality control, indicating possible interference with the internal control. These samples were all punches from the head of the strip, and they were excluded in the results shown in Fig. 2A–C. The protein-wise heatmap shows overall higher values using the punch method versus the tear fluid eluates (Fig. 2A). Protein detectability was significantly higher for the punch method (76% ± 6%) compared to the elution method (66% ± 5%; Fig. 2B; p ≤ 0.001). When analysing the different locations of the Schirmer strip, the superiority of the punch method remained consistent. (A: p = not applicable; B: p = 0.028; C: p = 0.006; D: p = 0.002; Fig. 2C).

A Student’s t-test test was used in (B) and (C), while a one-way ANOVA with Tukey's post hoc analysis was used to calculate the p values in (C) and (D). n = 20 for Fig. 2B, n = 5 for Fig. 2C,D.

### Protein composition of different locations of the Schirmer strip

The total protein concentration of the tear fluid eluates of different locations is shown in Fig. 2D. Similar total protein concentrations were seen between the Schirmer strip locations (A = 300 ± 105 µg/mL, B = 299 ± 123 µg/mL, C = 279 ± 97 µg/mL, D = 283 ± 71 µg/mL: p = 0.981). Location A of the strips showed overall slightly higher protein expression levels of the 92 proteins tested compared to the remaining locations of the strip for the punches (A: 5.82 ± 0.04 normalized protein expression (NPX); B: 4.91 ± 0.24 NPX; C: 4.65 ± 0.25 NPX; D: 4.58 ± 0.21 NPX: p < 0.001). In the eluates the difference was not statistically significant (A: 4.13 ± 0.33 NPX; B: 3.96 ± 0.41 NPX; C: 3.80 ± 0.20 NPX; D: 3.66 ± 0.27 NPX: p = 0.134).

**Figure 3:**
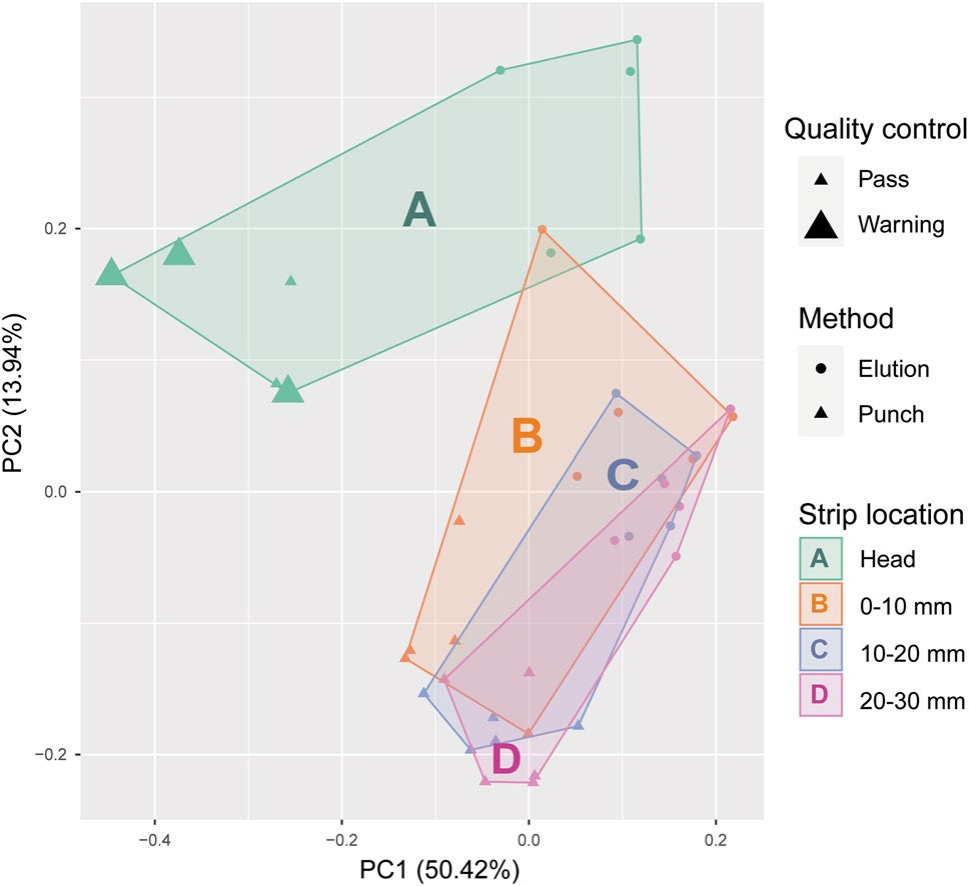
Principal component analysis of the protein composition (92 proteins) of different locations of the strips using the punch versus elution method. Scatterplot of the first two principal components based on Z-score scaled protein composition of all samples (n = 20*2) measured by the Olink Target 96 Inflammation panel. Location A (head of the strip) groups differently than Schirmer strip locations B, C and D, most noticeably the punch method location A groups separately. The samples with bold symbols provided a warning on the Olink proteomics quality control, which reflects the possible interference of proteins in these samples with the internal control.

A principal component analysis was performed of the protein composition in the two methods and the different locations of the Schirmer strip (Fig. 3). Here, the samples that did not pass the quality control are also shown. Location A clusters differently than the remaining locations of the strip. Moreover, the punch method samples group slightly different than the elution method samples.

### Influence of molecular weight on protein migration

**Figure 4:**
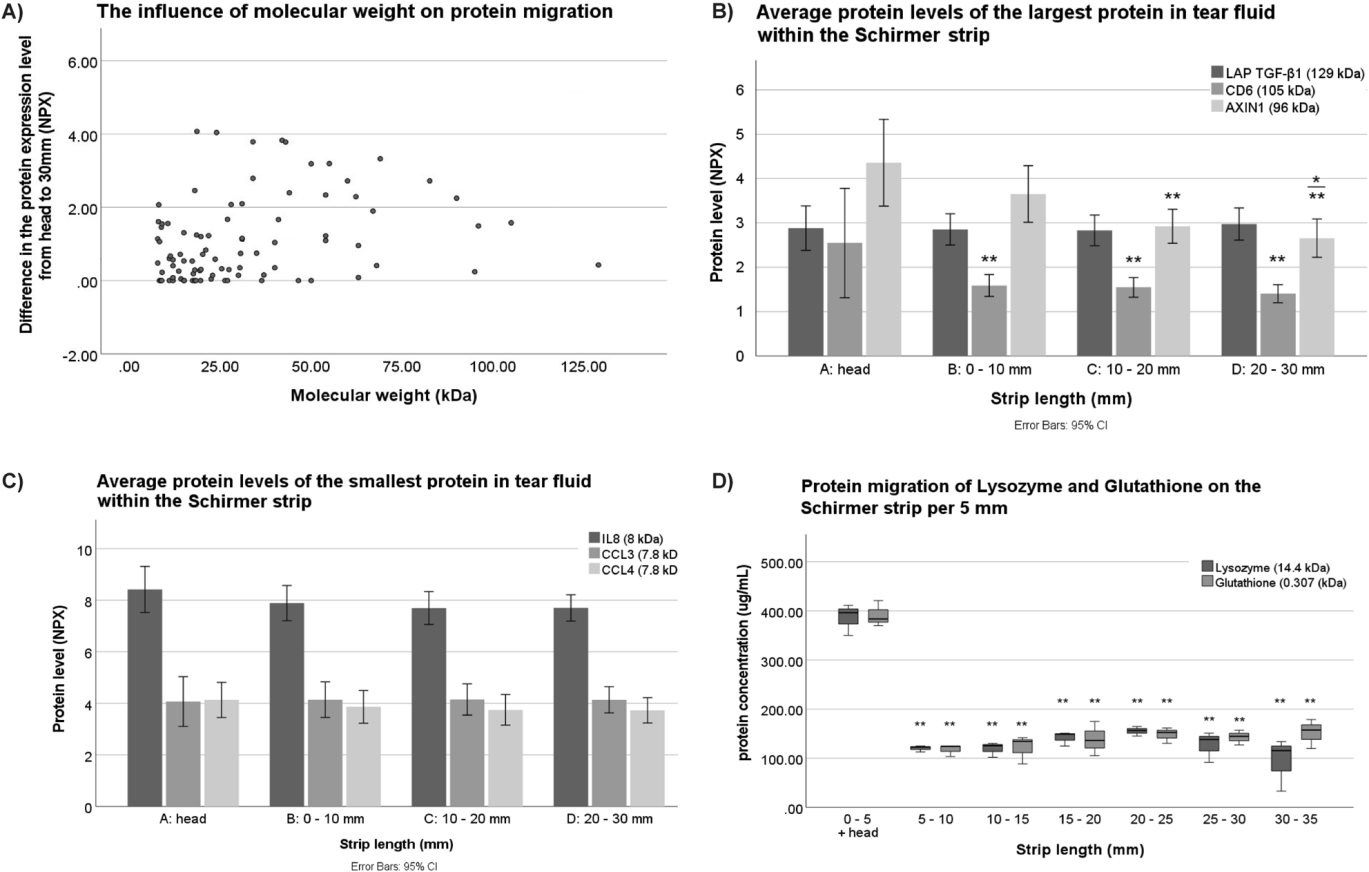
The influence of the molecular weight on protein migration over tear fluid Schirmer strips. (**A**) Scatterplot of the difference in protein expression level (NPX) of the head, location A, compared to location D**,** against the protein molecular weight. (Pearson rho = 0.227; p = 0.009). (**B**) Significant differences were found in the protein migration of cluster of differentiation 6 (CD6) (location A vs B: p = 0.001; A vs C: p = 4.72 ∙ 10^–4^; A vs D: p = 1.27 ∙ 10^–4^), and Axin-1 (location A vs C : p = 0.006; A vs D: p = 0.001; B vs D: p = 0.034). (**C**) No significant differences were found in the protein migration over the strip of interleukin-8 (IL8), chemokine ligand 3 (CCL3), and chemokine ligand 4 (CCL4). (**D**) The protein migration of spiked lysozyme (14.4 kDa) and glutathione (0.307 kDa) is displayed in the boxplots. A significant difference in protein concentration was found with respect to the migration of both lysozyme (head–5 mm vs 5–10 mm: p = 2.29∙10^–7^; head–5 mm vs 10–15 mm: p = 2.19∙10^–7^; head–5 mm vs 15–20 mm: p = 6.63∙10^–7^; head–5 mm vs 20–25 mm: p = 0.1∙10^–5^; head–5 mm vs 25–30 mm: p = 3.21∙10 ^-7^; head–5 mm vs 30–35 mm: p = 6.91∙10^–8^) and glutathione (head–5 mm vs 5–10 mm: p = 3.12∙10^–8^; head–5 mm vs 10–15 mm: p = 3.83∙10^–8^; head–5 mm vs 15–20 mm: p = 8.99∙10^–8^; head–5 mm vs 20–25 mm: p = 1.45∙10^–7^; head–5 mm vs 25–30 mm: p = 1.11∙10^–7^; head–5 mm vs 30–35 mm: p = 1.78∙10^–7^).

The influence of molecular weight on the protein migration is shown in Fig. 4. Proteins with higher molecular weight generally migrated less over the Schirmer strip, since the protein expression levels of the head of the strip (location A) were higher compared to the end of the strip (location D). This phenomenon of migration was significant, however not strong (r = 0.227, p = 0.009; Fig. 4A). The three heaviest proteins in the analysis were latency-associated peptide transforming growth factor beta (LAP TGFß; 129 kDa), cluster of differentiation 6 (CD6;105 kDa), and Axin-1 (96 kDa). No difference in protein expression level of LAP TGF-Beta of the different locations of the strip was detected (P = 0.926) (Fig. 4B), which was shown by both methods. However, a significant difference was observed in the protein migration of CD6 and Axin-1. The protein expression level of CD6 was significantly higher in the head compared to the other locations of the Schirmer strip (location A vs B: p = 0.001; A vs C: p = 4.72 ∙ 10^–4^; A vs D: p = 1.27 ∙ 10^–4^). Protein expression level in Axin-1 runs down from location A until location D (location A vs C: p = 0.006; A vs D: p = 0.001, B vs D: p = 0.034) (Fig. 4B). The three lightest proteins examined were interleukin-8 (IL8; 8 kDa), chemokine ligand 3 (CCL3; 7.8 kDa), and chemokine ligand 4 (CCL4; 7.8 kDa). No significant differences were found in the protein migration of these proteins. (Fig. 4C). To evaluate the influence of molecular weight on protein migration using a different approach, two proteins with various molecular weight, lysozyme (14.4 kDa) and glutathione (0.31 kDa), were spiked on independent Schirmer strips. The total protein concentration differed significantly between the first 5 mm and the following strip lengths up to 30 mm for both the proteins, while the protein concentration was similar throughout the remaining Schirmer strip (Fig. 4D).

Protein expression levels were measured by the Target 96 Inflammation panel in 4A, 4B, and 4C, while protein concentrations of spiked proteins were measured by the BCA assay in Fig. 4D. One-way ANOVA with Tukey’s post-hoc test was applied for differences in protein concentrations between the locations. Differences between the proteins were analysed using an independent samples *t*-test without variances using Welch correction. Error bars represent 95% confidence intervals. n = 5 for Fig. 4A–C and n = 3 for Fig. 4D.

### Intracellular proteins

We hypothesized that location A of the strip might contain cellular remnants and therefore increased intracellular proteins. In the analyzed panel of 92 proteins, 10 proteins were intracellular proteins, which indeed, in comparison to extracellular proteins, showed a significantly higher difference in protein NPX level in the head of the strip compared to the end. (p = 0.005; Student’s *t-*test). However, they also had a slightly higher average molecular weight compared to extracellular proteins (48 kDa vs 30 kDa; p = 0.03; Student’s *t-*test), which might be a confounding factor.

### Proteins with altered migration

The top 10 proteins with the highest difference in protein expression level between the head and the end of the strip were FMS-like tyrosine kinase 3 ligand (Flt3L), interleukin-18, adenosine deaminase (ADA), T-cell surface glycoprotein CD8 alpha chain (CD8A), NAD-dependent protein deacetylase sirtuin-2 (SIRT2), hepatocyte growth factor (HGF), caspase 8 (CASP8), STAM-binding protein (STAMBP), Sulfotransferase 1A1 (ST1A1), and tumor necrosis factor ligand superfamily member 14 (TNFSF14). The difference occurred mainly between the head of the strip and location B. Data on the migration capability of a specific protein is available in the supplementary material.

## Discussion

Tear fluid is a potential treasure for biomarker analysis in various eye and systemic conditions. However, the best method of pre-processing tear samples (before the specific protein determination) is not known. We show that Schirmer strips can be pre-processed using the simple punch method, which was superior to the elution method, before analyzing the proteome. The head of the strip, which is in contact with the conjunctiva during sampling, should be studied with caution. Protein expression levels over the remaining strip locations are similar.

Proteomics studies using tear fluid are highly expanding, as several analysis methods can nowadays deal with small samples’ volumes^4,10,21,22^. Next to the widely used mass spectrometry (MS), PEA technology is an attractive option. Using MS, a high number of proteins could be detected in tear fluid^5,13,16,23,24^, however, the pre-analysis remains time-consuming, deep profiling requires a depletion step, and interesting chemokines/cytokines that are less abundant remain hard to detect. Also, exact quantification remains an issue^23–25^. Using an antibody-based assay, the panel of biomarkers of interest can be chosen, and only a few microliters of sample are required^22^. PEA technology can evaluate a much greater number of proteins, compared to other multiplex techniques, but so far, very few studies have used this method studying tear fluid^18,22,26,27^, and little is known about the optimal pre-processing method, which is crucial for between-study comparisons.

Most often, tear proteins are eluted from the Schirmer strip before analyses. The idea of directly using punches for proteomic analysis came from the experience with dry blood spots^28^. To the best of our knowledge, the punch method has been used once with Schirmer strips to study metabolomics of tear fluid by Dammeier et al.^29^ In our experience, taking 1.2 mm diameter punches from the Schirmer strips was a highly simple and rapid procedure. Also, this enables the usage of the rest of the strip for other analyses or validation experiments. Our study showed higher NPX values and protein detectability using the punch method compared to the elution method for all locations of the strip, which might be explained by the absence of extraction loss, a dilution step, and the multiple sample transfer steps required in the elution method^14,19,20,30,31^. A mean protein detectability of around 70% was fairly high. Csosz et al. found that 45% of proteins of the Olink inflammation panel were present in > 75% of their samples. In that study, 11 out of 184 proteins (of multiple panels) could not be detected in tears, of which interleukin (IL)-2, IL-2RB, IL-20, IL-22RA1, IL-24, fibroblast growth factor 5, interferon gamma, signalling lymphocytic activation molecule, tumour necrosis factor and thymic stromal lymphopoietin were also not detected in our series^32^.

The proteome of the Schirmer strip head differed from the rest of the strip in our study. The study by Arslan et al. also identified a slightly higher number of proteins in the head of the strip (1153 proteins) compared to the rest of the strip (1107 proteins) using MS, while some proteins were solely identified in the head of the strip (246 proteins)^23^. We hypothesize this might be due to the presence of cellular remnants of the conjunctival epithelium, which could interfere with the internal control in the Olink analysis. However, this is subject for further studies.

When applying the punch method, it is crucial to know which location of the strip is representative. We found that protein expression level is higher in the head of the strip, which is correlated to the molecular weight of the proteins. The correlation coefficient however was small, and other factors, such as hydrophobicity, hydrophilicity, protein charge, and the formation of aggregates of proteins, might also be of influence. A study by Denisin et al. showed a significant, but small correlation between molecular weight, as well as hydrophobicity and in-strip retention of proteins during the elution step, and no significant correlation with protein charge^20,33^. In contrast, protein composition were similar over the other locations of the strip. No restriction by cellulose fibers of the Schirmer strip or a chromatography effect was seen in our study.

Our study covered a specific set of 92 inflammatory proteins, and extrapolation to not tested proteins remains uncertain. Regarding the influence of molecular weight, it is important to note that one of the heaviest proteins in our study is 105 kDa, which showed some restriction of migration from head to the remainder of the strip. In a study by Arslan et al. a 641 kDa protein was solely found in the head of the strip. It is possible that the effect of molecular weight might be slightly underestimated in our study^23^. We used phosphate buffered saline (PBS) as an extraction buffer in the elution method, while elution in ammonium bicarbonate containing 50 mM NaCl might gain a higher extraction rate and identification of proteins by MS, as shown in a study by Aass et al.^31^. However, no comparison with PBS was made in this study, and to what extent the ammonium buffer could interfere with the PEA analysis is yet unknown.

To conclude, we highlight the use of tear fluid samples in biomarker research and show that tear fluid samples could be studied with PEA technology, using the simple punch method of the Schirmer strip for its pre-processing. We encourage using one method and one location consistently and use internal control samples to allow comparisons of results in future studies.

## Methods

### Sample collection

Schirmer strips (True Blue Optics) containing tear fluid from 5 healthy participants without any history of eye diseases or dryness were collected from both eyes under comparable conditions. The samples were collected by the Maastricht Tear Fluid Biobank, University Eye Clinic Maastricht, Maastricht University Medical Centre (MUMC+), and Erasmus MC, University Medical Centre Rotterdam, the Netherlands. The head of the Schirmer strip was placed behind the lower eyelid at approximately 2/3th from the medial cantus. After 5 min, the strip was removed using disposable tweezers, and placed in an Eppendorf or Cryovial tube. The migration length in millimetres was reported, and the samples were stored at – 80 °C until further processing. The local Ethics Committee of both University medical centres (Medical Ethics Review Committee Erasmus MC and Medical Ethics Review Committee azM/UM) approved the study protocols and all methods were performed in accordance with the Tenets of the Declaration of Helsinki and its later amendments. All subjects gave written informed consent before tear fluid collection.

### Tear fluid pre-processing methods

Schirmer strips from 5 healthy participants were selected (one eye randomly selected per participant, all with tear migration length > 30 mm). The strips were cut longitudinally into two equal portions, one part was used for the punch method, and the other part for the elution method. The experimental set-up is shown in Fig. 1.

In the punch method, out of one half of the Schirmer strip, punches of 1.2 mm diameter using a biopsy punch tool (Tisch Scientific, Cleves, OH, VS) were taken from 4 locations of the strip (location A: head; location B: 0–10 mm; location C: 10–20 mm; location D: 20–30 mm), with sterilization of the biopsy punch tool between sampling. The punches were directly transferred to the Olink incubation buffer for analysis.

In the elution method, one half of the Schirmer strips was first cut into 4 locations (location A: head; location B: 0–10 mm; location C: 10–20 mm; location D: 20–30 mm), and subsequently cut into small pieces (approximately 1 mm) with sterilized scissors. The strips were eluted using a piggy-bag method described previously^9^. In short, the pieces were submerged in 60 µl extraction buffer PBS pH 7.4 with Complete mini protease inhibitor (Sigma Aldrich, Burlington, USA). The samples were incubated on a thermomixer for 1.5 h at 4 °C and 900 rpm. Thereafter, the strip pieces were transferred to a 0.5 mL Eppendorf tube with a syringe needle-punctured hole at the tip. The 0.5 mL Eppendorf tube was placed in the original Eppendorf tube, and centrifuged at 13,000 rpm for 1 min at 4 °C to obtain the eluates. 1 out of 40 µl of all eluates was supplied for PEA analysis.

### Total protein measurement (BCA)

The total protein content of tear fluid eluates was determined using the bicinchoninic acid (BCA) Protein Assay Kit (Pierce™, Thermo Fisher Scientific, Waltham, USA) according to the manufacturer’s instructions. Absorbance was measured at 562 nm on a microplate reader (CLARIOstar PLUS, BMG Labtech, Germany). Total protein concentration measurements of location A of the strips (head) were corrected for surface area, wherein the BCA result of locations A of the strip were multiplied with a correction factor. The correction factor was calculated with the following formula (surface area half circle/ surface are half square = $2{r}^{2}$/$\frac{1}{2}\pi {r}^{2}$ = 4/π).

### Targeted proteomics using proximity extension assay (PEA)

Punches of the Schirmer strip as well as 1 µl of tear fluid eluates were measured with the Olink Target 96 inflammation panel (product number: 95302) using the PEA technology (Olink, Uppsala, Sweden). A full list of 92 included inflammatory proteins is available in the supplementary material. In PEA, matched pairs of antibodies carry a unique DNA tag that will bind to the respective target protein in the tear sample. The matched pair of antibodies with their DNA are able to hybridize when brought in proximity. The hybridized tags are extended to an amplicon, and subsequently detected and quantified using quantitative PCR (qPCR). The number of qPCR cycles is related to the expression of the protein in the tear sample, shown in log base-2 NPX values. The technology uses 4 internal controls, incubation, extension, and detection control, that are spiked into each sample and well. They are used for normalization and evaluation of sample and run quality. Samples pass quality control if internal incubation and detection control are within ± 0.3NPX of the plate median. Protein detectability (%) was defined as the number of detectable proteins (having values above the lower limit of detection) from the total number of proteins tested (92).

### Protein distribution of spiked proteins within a Schirmer strip

On the head of the Schirmer strip 35μL of either 2 mg/mL lysozyme (Roche Diagnostics GmbH, Mannheim, Germany), or glutathione (VWR international BV, Amsterdam, The Netherlands) in 1× PBS was pipetted and then stored at − 80 °C. After storage, the entire strip was horizontally cut into 5 mm pieces which were separately eluted following previously noted protocol. The protein content of each 5 mm strip location was quantified using the BCA assay. Each condition was repeated in triplicate.

### Data analysis

Statistical analysis was performed using IBM SPSS Statistics 27. Descriptive statistics are shown in boxplots, as mean ± standard deviation (SD), or as mean and 95% confidence interval (CI). To assess statistical significance between variables, a Student’s *t*-test, or one-way ANOVA were applied. Olink NPX values were loaded into R (> = 4.2.2) and a Principal Component Analysis (PCA) was performed using “prcomp” on z-score scaled NPX values. A p-value below 0.05 was considered statistically significant, and is marked with an asterisk in the graphs. A double asterisk indicates a p-value below 0.01.

## Supplementary Information

Supplementary Information 1.

Supplementary Information 2.

## Data Availability

Supplementary data is available, and all other data underlying this article will be shared upon reasonable request to the corresponding author.
