# Supplementary material for: Evaluation of pre-processing methods for tear fluid proteomics using proximity extension assays
Source: Sci Rep. 2023 Mar 17;13:4433. doi: 10.1038/s41598-023-31227-1 (PMC10023677; doi:10.1038/s41598-023-31227-1)
Supplement: Supplementary file 2 — Supplementary Information 2. [file 41598_2023_31227_MOESM2_ESM.pdf]

Olink®

Protein assay list

Olink® Target 96 Inflammation

Product number: 95302

Adenosine Deaminase (ADA)	P00813	Fibroblast growth factor 23 (FGF-23)	Q9GZV9
Artemin (ARTN)	Q5T4W7	Fibroblast growth factor 5 (FGF-5)	Q8NF90
Axin-1 (AXIN1)	O15169	Fibroblast growth factor 19 (FGF-19)	O95750
Beta-nerve growth factor (Beta-NGF)	P01138	Fms-related tyrosine kinase 3 ligand (Flt3L)	P49771
Caspase-8 (CASP-8)	Q14790	Fractalkine (CX3CL1)	P78423
C-C motif chemokine 3 (CCL3)	P10147	Glial cell line-derived neurotrophic factor (GDNF)	P39905
C-C motif chemokine 4 (CCL4)	P13236	Hepatocyte growth factor (HGF)	P14210
C-C motif chemokine 19 (CCL19)	Q99731	Interferon gamma (IFN-gamma)	P01579
C-C motif chemokine 20 (CCL20)	P78556	Interleukin-1 alpha (IL-1 alpha)	P01583
C-C motif chemokine 23 (CCL23)	P55773	Interleukin-2 (IL-2)	P60568
C-C motif chemokine 25 (CCL25)	O15444	Interleukin-2 receptor subunit beta (IL-2RB)	P14784
C-C motif chemokine 28 (CCL28)	Q9NRJ3	Interleukin-4 (IL-4)	P05112
CD40L receptor (CD40)	P25942	Interleukin-5 (IL5)	P05113
CUB domain-containing protein 1 (CDCP1)	Q9H5V8	Interleukin-6 (IL6)	P05231
C-X-C motif chemokine 1 (CXCL1)	P09341	Interleukin-7 (IL-7)	P13232
C-X-C motif chemokine 5 (CXCL5)	P42830	Interleukin-8 (IL-8)	P10145
C-X-C motif chemokine 6 (CXCL6)	P80162	Interleukin-10 (IL10)	P22301
C-X-C motif chemokine 9 (CXCL9)	Q07325	Interleukin-10 receptor subunit alpha (IL-10RA)	Q13651
C-X-C motif chemokine 10 (CXCL10)	P02778	Interleukin-10 receptor subunit beta (IL-10RB)	Q08334
C-X-C motif chemokine 11 (CXCL11)	O14625	Interleukin-12 subunit beta (IL-12B)	P29460
Cystatin D (CST5)	P28325	Interleukin-13 (IL-13)	P35225
Delta and Notch-like epidermal growth factor-related receptor (DNER)	Q8NFT8	Interleukin-15 receptor subunit alpha (IL-15RA)	Q13261
Eotaxin (CCL11)	P51671	Interleukin-17A (IL-17A)	Q16552
Eukaryotic translation initiation factor 4E-binding protein 1 (4E-BP1)	Q13541	Interleukin-17C (IL-17C)	Q9P0M4
Fibroblast growth factor 21 (FGF-21)	Q9NSA1	Interleukin-18 (IL-18)	Q14116

Table continues on reverse ▶

Interleukin-18 receptor 1 (IL-18R1)	Q13478	Programmed cell death 1 ligand 1 (PD-L1)	Q9NZQ7
Interleukin-20 (IL-20)	Q9NYY1	Protein S100-A12 (EN-RAGE)	P80511
Interleukin-20 receptor subunit alpha (IL-20RA)	Q9UHF4	Signaling lymphocytic activation molecule (SLAMF1)	Q13291
Interleukin-22 receptor subunit alpha-1 (IL-22 RA1)	Q8N6P7	SIR2-like protein 2 (SIRT2)	Q8IXJ6
Interleukin-24 (IL-24)	Q13007	STAM-binding protein (STAMBP)	O95630
Interleukin-33 (IL-33)	O95760	Stem cell factor (SCF)	P21583
Latency-associated peptide transforming growth factor beta-1 (LAP TGF-beta-1)	P01137	Sulfotransferase 1A1 (ST1A1)	P50225
Leukemia inhibitory factor (LIF)	P15018	T cell surface glycoprotein CD6 isoform (CD6)	Q8WWJ7
Leukemia inhibitory factor receptor (LIF-R)	P42702	T-cell surface glycoprotein CD5 (CD5)	P06127
Macrophage colony-stimulating factor 1 (CSF-1)	P09603	T-cell surface glycoprotein CD8 alpha chain (CD8A)	P01732
Matrix metalloproteinase-1 (MMP-1)	P03956	Thymic stromal lymphopoietin (TSLP)	Q969D9
Matrix metalloproteinase-10 (MMP-10)	P09238	TNF-beta (TNFB)	P01374
Monocyte chemotactic protein 1 (MCP-1)	P13500	TNF-related activation-induced cytokine (TRANCE)	O14788
Monocyte chemotactic protein 2 (MCP-2)	P80075	TNF-related apoptosis-inducing ligand (TRAIL)	P50591
Monocyte chemotactic protein 3 (MCP-3)	P80098	Transforming growth factor alpha (TGF-alpha)	P01135
Monocyte chemotactic protein 4 (MCP-4)	Q99616	Tumor necrosis factor (Ligand) superfamily, member 12 (TWEAK)	O43508
Natural killer cell receptor 2B4 (CD244)	Q9BZW8	Tumor necrosis factor (TNF)	P01375
Neurotrophin-3 (NT-3)	P20783	Tumor necrosis factor ligand superfamily member 14 (TNFSF14)	O43557
Neurturin (NRTN)	Q99748	Tumor necrosis factor receptor superfamily member 9 (TNFRSF9)	Q07011
Oncostatin-M (OSM)	P13725	Urokinase-type plasminogen activator (uPA)	P00749
Osteoprotegerin (OPG)	O00300	Vascular endothelial growth factor A (VEGF-A)	P15692

For more details visit www.olink.com/inflammation

www.olink.com

For research use only. Not for use in diagnostic procedures.
This product includes a license for non-commercial use. Commercial users may require additional licenses. Please contact Olink Proteomics AB for details.
There are no warranties, expressed or implied, which extend beyond this description. Olink Proteomics AB is not liable for property damage, personal injury, or economic loss caused by this product.
Olink® is a registered trademark of Olink Proteomics AB.

© 2017–2022 Olink Proteomics AB. All third party trademarks are the property of their respective owners.

Olink Proteomics, Dag Hammarskjölds väg 52B , SE-752 37 Uppsala, Sweden
1029, v2.0, 2022-06-14
